# The Impact of Air Pollution on the Course of Cystic Fibrosis: A Review

**DOI:** 10.3389/fphys.2022.908230

**Published:** 2022-06-02

**Authors:** Marion Blayac, Patrice Coll, Valérie Urbach, Pascale Fanen, Ralph Epaud, Sophie Lanone

**Affiliations:** ^1^ Univ Paris Est Creteil, INSERM, IMRB, Creteil, France; ^2^ Université Paris Cité and Univ Paris Est Créteil, CNRS, LISA, Paris, France; ^3^ AP-HP, Hopital Henri-Mondor, Service Génétique, Creteil, France; ^4^ Centre Hospitalier Intercommunal, Centre des Maladies Respiratoires Rares (RespiRare®)-CRCM, Creteil, France

**Keywords:** cystic fibrosis, air pollution, environmental factors, acute exposure, chronic exposure, CF models

## Abstract

Cystic fibrosis (CF) is a lethal and widespread autosomal recessive disorder affecting over 80,000 people worldwide. It is caused by mutations of the *CFTR* gene, which encodes an epithelial anion channel. CF is characterized by a great phenotypic variability which is currently not fully understood. Although CF is genetically determined, the course of the disease might also depend on multiple other factors. Air pollution, whose effects on health and contribution to respiratory diseases are well established, is one environmental factor suspected to modulate the disease severity and influence the lung phenotype of CF patients. This is of particular interest as pulmonary failure is the primary cause of death in CF. The present review discusses current knowledge on the impact of air pollution on CF pathogenesis and aims to explore the underlying cellular and biological mechanisms involved in these effects.

## Introduction

Cystic Fibrosis (CF) is a monogenic disease caused by pathogenic mutations in the Cystic Fibrosis Transmembrane conductance Regulator (*CFTR*) gene which encodes the CFTR protein, an anion channel responsible for chloride and bicarbonate ion transport across epithelial cells. For a more complete description of CF disease please, refer to Shteinberg et al. (2021). CFTR also regulates many other mechanisms in epithelial physiology and has been reported to inhibit the epithelial Na^+^ channel (ENaC) activity in the airways. Dysfunctional CFTR results in dehydration of airway surface liquid (ASL) and is associated with persistent airway infection and sustained inflammation, leading to obstructive lung disease and progressive structural damage (Boucher, 2007). While CF is a systemic disease characterized by altered lung mucociliary clearance, exocrine pancreatic insufficiency, intestinal obstruction and infertility, the bronchopulmonary disease remains the main driver of the high morbidity and early mortality of CF patients (Elborn, 2016). The progression of the disease mainly depends on pulmonary exacerbations defined as acute deterioration of respiratory symptoms which ultimately impair lung function and quality of life (Ferkol et al., 2006; Stanford et al., 2021). These exacerbations’ episodes, most frequently caused by lung bacterial infections, include increased cough, increased sputum production, increased use of antibiotics, dyspnea, and decreased lung function. Repeated exacerbation events, chronic infection and persistent inflammation lead to progressive and irreversible lung damage and respiratory failure (Sanders et al., 2010, 2011; de Boer et al., 2011; Waters et al., 2012).

CF patients present a great genotypic variability with more than 2,000 *CFTR* gene mutations identified so far (Rommens, 2011). These mutations are classified into six classes based on their effect on CFTR synthesis and function (Elborn, 2016). Each of these mutations occurs at a variable frequency (Lao et al., 2003; De Boeck et al., 2014) with a heterogenous geographical distribution (Mirtajani, 2017). Class I–III mutations are usually associated with a more severe CF disease prognosis as CFTR protein function is totally suppressed. Class IV–VI mutations are characterized by a residual CFTR function and usually cause a disease of more moderate severity (Elborn, 2016). Interestingly, while CFTR genotype correlates well globally with the pancreatic phenotype, it mostly fails to predict pulmonary-disease severity (Burke et al., 1992; The Cystic Fibrosis Genotype-Phenotype Consortium, 1993; Kerem and Kerem, 1996; Duguépéroux and De Braekeleer, 2004; Cutting, 2015; Raynal and Corvol, 2020). Twin and sibling studies have shown a smaller difference in lung function between monozygotic twins than between dizygotic twins or siblings (Collaco et al., 2010). Such difference appears to increase when twins and siblings start living apart and stop sharing common environment. This phenotypic variability suggests the involvement of other contributors especially to the CF airway disease, unrelated to CFTR itself. Several genes (i.e., *NOS*, *TGFB1*, *MBL2*, *MUC4/MUC20, SLC9A3, SLC6A14* or *HLA* Class II) have been identified as possible gene modifiers of CF lung disease (Corvol et al., 2015; Sepahzad et al., 2021). The splicing machinery could also modify CF severity (Nissim-Rafinia and Kerem, 2005). Beside these genetic and epigenetic modifiers, non-genetic factors also appear as relevant modifying contributors to CF pathophysiology; environmental factors (Szczesniak et al., 2020) such as tobacco smoke (Campbell et al., 1992; Kopp et al., 2016), air pollution (Brugha et al., 2018), climate and seasonal changes (Ramsay et al., 2016) or socioeconomic status (Oates and Schechter, 2016). This review particularly focuses on the contribution of air pollution.

**FIGURE 1:**
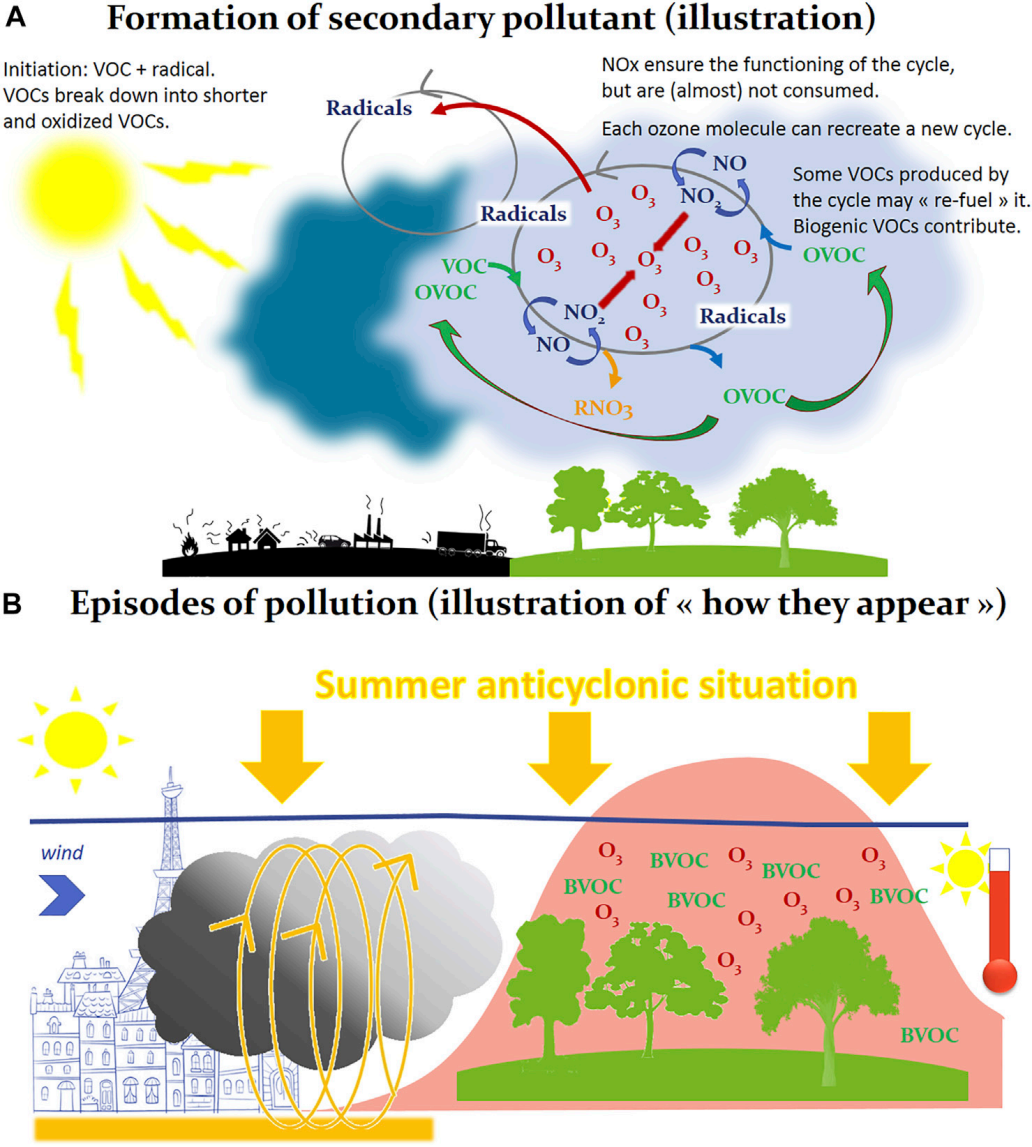
Formation of secondary pollutants **(A)** and episodes of pollution **(B)**. In addition to primary pollutants (gases or particles, emitted naturally or by anthropogenic activity), many so-called “secondary” pollutants are not emitted but formed from other pollutants, as it is the case in Panel 1A in this illustration of the production of ozone (O_3_) in the gas phase. Here the formation of ozone results from reactions cycles between nitrogen oxides (NO/NO_2_) and VOCs, in the chemical reactor constituted by the atmosphere, these chemical reactions using solar energy as energy source. Atmospheric radicals are the intermediary species involved in these chemical cycles. All this explains how, with favorable conditions, very large quantities of ozone can be formed in a few hours of sunshine, downwind of cities. An illustration of such phenomenon is reported in Panel 1B, where we are facing a summer anticyclonic situation, the pollutants emitted for example in the city (left section of 1B) react in the urban atmosphere under the sunlight. Under the effect of the wind, this primary pollution can migrate to peri-urban or rural areas, cross other chemical compounds (as Biogenic Volatile Organic Compounds, BVOC, emitted by trees) and generate a secondary pollution, symbolized in Panel 1B by a production of ozone (O_3_, pollution peak). We are in the case of a secondary pollution at the regional scale, spatially extended, which will present a maximum in the afternoon because the sunshine irradiation, and consequently the intensity of the photochemistry, are maximum.

Outdoor air pollutants are numerous (several hundreds to several thousands of chemical species), for most of them at trace levels (from few ppb—parts per billion, to ppm—parts per million), and are gaseous (nitrogen dioxide—NO_2_, carbon dioxide—CO_2_, methane—CH_4_, etc.) but also under particular phases: these atmospheric particles, or *aerosols*—also referred to as particulate matter—are a suspension of liquid or solid in the gas phase and are categorized according to their size—coarse particles (PM_10_), fine particles (PM_2.5_) and ultrafine particles (PM_0.1_). Pollutants can be divided into two groups related to their origin: 1) primary pollutants which are air pollutants emitted directly from a source—as an illustration nitrogen oxides (NO_X_) or soot particles mainly emitted from traffic; 2) secondary pollutants that are not directly emitted, but are formed when other pollutants react in the lower atmosphere—as an illustration the production of the oxidant ozone (O_3_) resulting from reactions between nitrogen oxides (NOx) and volatile organic compounds (VOCs) (Figure 1A) (Walker, 1977). These numerous chemical compounds, gases and aerosols, react continuously in the atmosphere under solar irradiation, and are also stirred and transported in the atmosphere (Holton et al., 1995). Thus, the composition of the atmosphere varies according to time and space (in the order of few seconds and few meters for OH^.^, and of 10 years and 1.000 km for CH_4_; illustrations and more extended descriptions may be found in (Wayne, 2000; Ravishankara, 2003). These well-known atmospheric composition and evolution are illustrated by Figures 1A,B. Much more details may be found in (Seinfeld and Pandis, 2016).

Public health concerns, with raising questions about the impact of air pollution on our health, has driven to monitor and predict air quality (as in the case of meteorology). Atmospheric measurements are carried out throughout the world, in a more or less intense way depending on the level of development of the countries. The number of measuring stations, and their distribution on the territories, answer to rules or laws (as an example in France, rules are stated at the European level). These rules are essential to guarantee a quality of measurement that is comparable from one region or country to another (Kracht et al., 2018). For example, in the Paris region, different types of measurements and analysis stations are spread over a 100 km radius around Paris and measure the quality of the air breathed by the population (more than 12 million inhabitants in the whole region), regardless of their environment, near or far from the sources of pollution. The network in charge of these measurements has about 70 measuring stations, including more than 50 permanent stations, acting as mini static laboratories (Rodriguez et al., 2019).

Automatic stations (measuring for example O_3_, NOx, sulfur dioxide—SO_2_, carbon monoxide—CO, etc.) ensure a continuous measurement every hour usually, and allow a real time follow-up of the air quality. The choice of the location of the automatic stations and of the types of the pollutants measured in these stations is primarily based on public health concerns. There are several types of stations: background stations (far from pollution sources, in particular traffic routes), near traffic stations (located near a road infrastructure as area representative of the maximum level of exposure for the population), urban stations (monitoring the average exposure of the population to air pollution phenomena known as “background” in urban centers), peri-urban stations (monitoring the photochemical pollution, in particular O_3_ and its precursors, and monitoring the average level of exposure of the population to “background” air pollution phenomena on the outskirts of the urban center), rural stations (monitoring of the exposure of ecosystems and of the population in rural areas to “background” air pollution, in particular photochemical pollution, at a regional scale) and industrial site stations (information on concentrations measured in areas representative of the maximum level to which the population near a stationary source is likely to be exposed by plume or accumulation phenomena). Such continuous monitoring allows to measure the level of exposure of individuals over different times scales, either acutely (referring to an exposure lasting from 1 day to a few days as during an air pollution episode) or chronically (referring to a continuous or repeated exposure over a long period of time, from months to years). In addition, some pollutants are not measured automatically and in real time by the stations but are measured by sampling followed by delayed analysis techniques in specific laboratories, or even by dedicated measurement field campaigns. Finally, automatic station network can be reinforced by measurements using diffusion tubes. This method guarantees a regular but not continuous monitoring and allows to calculate an annual average and to estimate the situation with regard to the regulatory values.

Numerical modeling (Chemistry Transport Models) is a complementary tool to pollutant emission measurements and inventories. It allows to estimate the concentrations of various pollutants at any point of a given geographical area, for past, present, or future periods. These numerical tools are based on the representation of the phenomena involved in the formation and fate of air pollution: chemical transformations, transport, dispersion, emissions, and depositions. These different numerical models operate at various temporal and geographical scales (from the street scale to the continental scale) and their use requires to characterize the parameters influencing the concentrations of pollutants (for example meteorology). Overall, the use of this mathematical tool supports air quality monitoring, as it allows to characterize the air pollution at any point of the represented geographical domain.

The challenge of measuring reactive trace species in the atmosphere (at ppt—parts per trillion to ppm levels), ideally on a continuous basis, and of integrating these data into the Chemistry Transport Models in order to characterize the exposure of populations at a given location over a given period of time, is therefore a very high-level challenge. This is the main barrier to linking the effects of air pollution to the observed health impacts on populations.

Amongst these hundreds of pollutants, some so-called “indicators” (such as O_3_, NO_2,_ SO_2_, PM_2.5_, and PM_10_) are under official monitoring in most countries and are expected to fit air quality guidelines. World Health Organization (WHO) estimates that 99% of people worldwide breath polluted air, with effects on health that are clearly established (World Health Organization, 2021). Accounting for 4.2 million premature deaths yearly worldwide (World Health Organization, 2021), ambient air pollution currently constitutes the major environmental threat for human health and ranks as the fourth leading risk factor for premature deaths (Health Effects Institute, 2020). Regarding respiratory effects, it is well documented that air pollution reduces lung function (Paulin and Hansel, 2016), worsens respiratory condition of patients with pre-existing pathologies such as asthma and chronic obstructive pulmonary disease (COPD) (Hoek et al., 2013; Thurston et al., 2020) and increases lung cancer incidence (Raaschou-Nielsen et al., 2013). Since 2013, air pollution has been classified as carcinogenic to humans by the International Agency for Research Cancer (IARC) (International Agency for Research on Cancer, 2013). In a recent paper, the loss of life expectancy from ambient air pollution was shown to be 2.9 years (Lelieveld et al., 2020). In addition, it is estimated that the mean life expectancy would increase by 1.7 years if all anthropogenic emissions were removed. For children, air pollution is associated with increased preterm birth and low birth weight (Stieb et al., 2012; Dadvand et al., 2013; Fleischer et al., 2014), reduced pulmonary growth and lung function (Gauderman et al., 2007; Hwang et al., 2015; Milanzi et al., 2018) together with an increased risk of developing respiratory diseases (Goldizen et al., 2016).

With known effects for healthy people as well as for people with pre-existing condition(s), air pollution could constitute an important contributing factor for CF disease. Moreover, in a context where air pollution is often above WHO’s recommended values and where CF patients live older (while being diagnosed at birth in most cases), it is of particular interest to consider air pollution and its possible harmful effects on the course of the disease. Strikingly, studies dealing with specific implication of air pollution on the course of CF airway disease are scarce and only quite recent. Our goal here is to review the actual knowledge on the role of air pollution in the phenotypic variability observed in CF as well as to highlight recent advances in the underlying mechanisms.

## Search Strategy

Studies of interest were identified by searching PubMed from 1990 to present with the term “cystic fibrosis” or “CFTR” combined with the specific terms “air pollution” or “particulate matter,” “ozone,” “nitrogen dioxide,” “sulfur dioxide,” “diesel” or “exhaust.” Key words search was limited to title and abstract. Human as well as mouse studies were considered. Among the 62 studies retrieved after this search, 14 were chosen based on their title and abstract. Studies were included if they assessed health outcomes in people with CF or in CF experimental models in relation with air pollution exposure measurement data or experimental exposure. Though conducted in non-CF airway cells and non-CF mouse model, two more studies were included because they investigated direct effects of pollutants on CFTR expression and/or function and airway fluid secretion. Finally, one additional study was identified when reviewing references of the articles initially selected. A total of 17 studies were overall included in this review (Tables 1, 2, 3).

**TABLE 1:** Summary and comparison of results obtained by epidemiological studies.

Study author/Year (References)	n (participants)	Exposure data-Pollutants concentration	Clinical outcome						
			Number of exacerbations		Lung function (as FEV_1_)		Primary infection		
Chronic exposure
Goss et al. (2004)	11 484	**PM**_**2.5**_ **= 13.7 (4.2)** **µg/m** ^**3**^	↗ (^a^)	OR = 1.21 (1.07–1.33) for PM_2.5_	↘ (^a^)	↘ FEV_1_ of 155 ml (115–194) for PM_2.5_	N.A	
		**PM**_**10**_ **= 24.8 (7.8)** **µg/m** ^**3**^		OR = 1.08 (1.02–1.15) for PM_10_				
		**O**_**3**_ **= 51 (7.3)** **ppb**		OR = 1.10 (1.03–1.17) for O_3_		↘ FEV_1_ of 38 ml (18–58) for PM_10_		
Jassal et al. (2013)	145	PM_2.5_ = 16.57 (13–17.6) µg/m^3^	↗ (^b^)	OR = 6.7 (1.23–54.49) for residence/road distance	N.A		N.A	
		O_3_ = 0.12 (0.11–0.14) ppm						
		**Residential proximity to a major roadway**						
Psoter et al. (2015)	3 575	**PM**_**2.5**_ **= 12.3 (2.7)** **µg/m** ^**3**^	N.A		N.A		↗ *P.aeruginosa* (^a^)		HR = 1.24 (1.01–1.51) for PM_2.5_
Psoter et al. (2017)	3 012–4 255	**PM**_**2.5**_ **= 12.31 (2.68)** **µg/m** ^**3**^	N.A		N.A		↗ MRSA (^a^)		HR = 1.56 (1.13–2.14) for PM_2.5_
Acute exposure
Goeminne et al. (2013)	215	**PM**_**10**_ **= 24 (17.6–32.1) µg/m** ^**3**^	↗ (^a^)	OR = 1.043 (1.004–1.084) for PM_10_	N.A		N.A	
		**O**_**3**_ **= 72.4 (57.3–90.3) µg/m** ^**3**^		OR = 1.106 (1.05–1.166) for O_3_				
		**NO**_**2**_ **= 23.9 (17.2–31.9) µg/m** ^**3**^		OR = 1.034 (1.003–1.067) for NO_2_				
Farhat et al. (2013)	103	PM_10_ = 42.03 (18.63)** **µg/m^3^	↗ (^c^)	RR = 1.86 (1.14–3.02) for O_3_	N.A		N.A	
		**O**_**3**_ **= 76.82 (36.81) µg/m** ^**3**^						
		NO_2_ = 103.02 (35.56)** **µg/m^3^						

PM, particulate matter; O_3,_ ozone; NO_2,_ nitrogen dioxide; FEV_1_, Forced Expiratory Volume in 1 s; MRSA, Methicillin-Resistant Staphylococcus Aureus; N.A, Not Available; Pollutants concentrations are presented as Median (Q1–Q3) or as Mean (SD). Pollutants in bold are those significantly associated with change in clinical outcome.

a Effects observed for a 10 μg/m^3^ or 10 ppb increase in pollutant concentration.

b Effects observed for a 1,000 m decrease in residential/major roadway distance.

c Effects observed for an interquartile range (45.62 μg/m^3^) increase in O_3_ concentration. OR, Odds-Ratio; RR, relative risk; HR, Hazard Risk. Ratios are presented with their 95% confidence interval in parentheses.

**TABLE 2:** *In vivo* experimental studies.

Study author/Year (References)	n	Animal model	Biological outcome	Pollutants	Exposure data
Qu et al. (2009)	—	Wistar rat	CFTR expression	O_3_	1-h per day 1.5 ppm O_3_ for 3 days
Shi et al. (2019)	6	BALB/c mice	Airway surface liquid secretion	Diesel particulate matter	0.5 mg, 10 μg/μl
			CFTR expression		
Geiser et al. (2014)	2–35	Cftr^tm1HGU^ mice	Lung function	TiO_2_ and CNP	1-h TiO_2_NP inhalation: 20 nm, 30–40 μg/m^3^
			Localization and biokinetics of NP		
			Inflammation		CNP Intratracheal instillation: 5–12 nm, 20 μg, 0.4 μg/μl
Geiser et al. (2013)	2–4	Scnn1b-Tg mice	Cellular uptake and localization of NP	Au NP	2-h inhalation
					AuNP, 21 nm, 1.2 mg/m^3^

O_3_, ozone;NP, nanoparticles; TiO_2_, titanium dioxide; C, carbon; Au, Gold.

**TABLE 3:** *In vitro* experimental studies.

Study author/Year (References)	n	Cell model	Biological outcome	Pollutants	Exposure data
Kamdar et al. (2008)	3	CF IB3-1 and WT S-9 human bronchial epithelial cell lines	Cellular viability	PM_2.5_	Exposure by aerosolization (1 h)
			Apoptosis		
			ROS production		25 μg/cm^2^
Ahmad et al. (2011)	3–6	ALI cultures of non-CF and CF cell lines and primary airway epithelial cell cultures derived from non-CF and CF patients	Cell survival	O_3_	100–1,000 ppb O_3_ for 4–18 h
			Pro-inflammatory cytokines		
Jeannet et al. (2016)	4	ALI cultures of normal and CF HBE cells and BEAS-2B bronchial epithelial cell line	Epithelial integrity	Ag and C NP	Exposure by aerosolization (36–3 600 s)
			Cell death		20 nm for both NP
			Pro-inflammatory mediators		AgNP: 2, 18, and 176 ng/cm^2^
					CNP: 0.3, 33, and 189 ng/cm^2^
Leni et al. (2020)	9	ALI cultures of normal and CF HBE cells	Cytotoxicity	PM_2.5_ and PM_10_	Water solubilization of PM filter extracts
			Pro-inflammatory mediators		Exposure for 4 h
			Gene expression		Low doses: 0.9–2.5 μg/cm^2^
					High doses: 8.8–25.4 μg/cm^2^
Künzi et al. (2015)	3–6	ALI cultures of normal and CF HBE cells and BEAS-2B bronchial epithelial cell line	Cytotoxicity	Aged gasoline exhaust particles (secondary organic aerosol)	2-h aerosolization
			Pro-inflammatory mediators		Particle dose deposited: 10–350 ng/cm^2^
Krapf et al. (2017)	3	ALI cultures of normal and CF HBE cells and BEAS-2B bronchial epithelial cell line	Cell death	Wood combustion particles (primary and secondary aerosols)	2-h aerosolization
			Oxidative markers		Deposited mass:
			Inflammatory response		- Primary: 133.2–199.5 ng/cm^2^
					- Secondary: 183.3–301.4 ng/cm^2^
Ahmad et al. (2012)	3–6	ALI cultures of non-CF and CF cell lines and primary airway epithelial cell cultures derived from non-CF and CF patients	NP deposition and uptake	Polystyrene NP and O_3_	24-h exposure to aerosolized NP (40 nm, 0.1 μg/cm^2^)
			Cell death and cellular integrity		
			Pro-inflammatory cytokine IL-8		200–500 ppb O_3_ for 8 h
Qu et al. (2009)	4–7	Normal HBE cells	CFTR expression	O_3_	1.5 ppm O_3_ for 30 min
			CFTR chloride current		
			Signal pathways		
Shi et al. (2019)	4	Calu-3 cells	Cell viability	Diesel particulate matter	100–400 μg/ml for 24–48 h
			Airway surface liquid secretion		
			CFTR expression		

ALI, Air-Liquid Interface; ROS, reactive oxygen species, PM: particulate matter; O_3,_ Ozone; Ag, Silver; C, carbon; NP, nanoparticles.

## Health Outcomes of Air Pollution Exposure in Cystic Fibrosis Patients

### Chronic Exposures

The first studies describing health outcomes of air pollution exposure in CF patients have focused on chronic exposures. There is only four of them so far. The very first study to address this issue has been published by Goss et al. (2004), in 2004, who surveyed a cohort of 11,484 patients during a 2 year-interval. In this study, the mean annual values for O_3_, NO_2_, SO_2_, CO, and particulate matter (PM) were considered. Authors linked the exposure data to pulmonary function, exacerbations and mortality data. Pulmonary function was estimated with forced expiratory volume in 1 s (FEV_1_) and exacerbations were defined as a “condition requiring hospitalization” or as “the at home use of intravenous antibiotics.” A negative linear association was found between lung function and particulate pollution (PM_2.5_ and PM_10_); for a 10 μg/m^3^ increase in PM_2.5_ and PM_10_, a significant decrease in FEV_1_ was observed (Table 1). No association was found with the other pollutants (O_3_, NO_2_, SO_2_, and CO). As for exacerbations, patients were divided into two groups according to the number of exacerbations experienced during the study period: none or one exacerbation versus two or more. The characterization of groups showed that the patients with more than two exacerbations were globally older, had a reduced lung function, a worse nutritional status and were more likely to be infected with the bacteria *Pseudomonas aeruginosa* (*P.aeruginosa*) and *Burkholderia cepacia* complex (*B.cepacia*) compared to those who suffer less than two exacerbations. Patients with two or more exacerbations also lived in regions with higher levels of PM_2.5_, PM_10,_ and O_3_. Authors showed that, for every 10 μg/m^3^ increase in PM_2.5_ and PM_10_ concentration, there was respectively a 21% and 8% higher risk of presenting more than two exacerbations (Table 1). Moreover, a 10-ppb increase in O_3_ concentration resulted in 10% increase in that same risk. No difference in the mean annual concentrations of NO_2_, SO_2_, and CO was observed between the two groups and no significant association with the exacerbation number was noted for these pollutants. Finally, although the result was not significant, a 10 μg/m^3^ increase in PM_2.5_ exposure substantially increased the mortality risk (32% higher risk).

In another study, Jassal et al. (2013) took in consideration the participant’s home distance to a major road or freeway in addition to O_3_ and PM_2.5_. The goal here was to use the proximity to roadways as a proxy for long-term traffic pollution, as concentrations of particles are elevated near roads due to emissions by vehicles exhaust. This 5-year retrospective study, dedicated to study the effects of air pollution on pulmonary exacerbation frequency, was conducted in 145 CF patients. Exacerbation and patient’s categorization were defined as in Goss’s study. No significant difference in O_3_ and PM_2.5_ levels could be found between the two exacerbation groups. Residential proximity to a roadway was however higher for individuals who experienced more than two exacerbations (Table 1). While the pollutant levels were similar between studies, the failure to replicate O_3_ and PM_2.5_ effects observed by Goss is probably due to a lack of statistical power in Jassal’s study. This latter study is however the first to describe the influence of proximity to traffic on the clinical condition of CF patients as opposed to classical quantitative measures of pollutants. Of note, proximity to traffic implies the consideration of pollutants other than the ones usually measured. Eventually, these results suggest that residential proximity to roadway could increase the susceptibility for acute exacerbation events in CF patients.

Bacterial colonization constitutes a central element of exacerbations and a key determinant of the severity of exacerbation episodes in CF. Psoter et al. (2015) formulated the hypothesis that exposure to air pollution could increase the risk of primary infection by *P.aeruginosa*. Although this micro-organism is the major respiratory pathogen in CF patients with an initial colonization usually occurring within the first 10 years of life, earlier *P.aeruginosa* infection, particularly before the age of five is strongly associated with a more severe CF lung disease later (Pittman et al., 2011). Psoter et al. (2017) therefore followed children under the age of 6 (3,575) and recorded the time to initial acquisition of *P.aeruginosa*, defined as the apparition of a first positive respiratory culture while on study. For exposure evaluation, the mean yearly PM_2.5_ concentration during the year preceding birth of each child was considered. During a mean follow-up period of 1.8 years, 1,711 (48%) children acquired *P.aeruginosa* with a median age of acquisition of 15 months. Exposure to PM_2.5_ was found to be slightly higher among children who acquired *P.aeruginosa* during the follow-up period compared to those who remained uninfected. In addition, a 10 μg/m^3^ PM_2.5_ concentration increase was significantly associated with a 24% increase in the risk of primary colonization by *P.aeruginosa* (Table 1). In a companion study based on the same cohort and experimental design, Psoter and colleagues evaluated if exposure to PM_2.5_ could be a risk factor for the initial acquisition of other respiratory pathogens in young CF children. They looked at the methicillin-sensitive (MSSA) and -resistant (MRSA) forms of *Staphylococcus aureus*, *Stenotrophomonas maltophilia* and *Achromobacter xylosoxidans*. Similar to the initial study, children who declared colonization during the follow-up period had a greater PM_2.5_ exposure compared to those who remained free of pathogens, independently of the pathogen considered. A 10 μg/m^3^ increase in PM_2.5_ exposure was associated with a 68% increased risk of MRSA acquisition but was not associated with the acquisition of other respiratory pathogens (Table 1).

### Acute Exposures

Only two studies so far have focused on the effects of acute exposure to pollution peaks on the course of CF disease. Goeminne et al. (2013) led a case-crossover retrospective study over a period of 12 years (Table 1). The goal was to explore the link between the short-term increase in PM_10_, O_3_, and NO_2_ and the onset of pulmonary exacerbations. An exacerbation was defined by the use of oral or intravenous antibiotics for respiratory event. Pollution levels were calculated for the last 2 days before and on the day of the exacerbation event. Data analysis showed an increase in the number of exacerbations on days with higher air pollution (Table 1). The authors found a significant association for a 10 μg/m^3^ increase in all three pollutants and same-day exacerbation onset. An overall increase in risk was also observed throughout the three measured days suggesting pollution peaks could have delayed effects. The strength of this study is its case-crossover design in which every subject is its own control, thus reducing the influence of confounding factors.

In a more size-limited study, Farhat et al. (2013) studied the effects of short-term variations in the concentrations of air pollutants. The study was conducted in the metropole of Sao Paulo on the occurrence of exacerbations in 103 CF children and teenagers for 1 year (Table 1). Exacerbation was defined as the presence of at least three of the following symptoms: fever, increase in sputum production or cough intensity, change in sputum color, worsening dyspnea, loss of appetite, >10% decrease of FEV_1_, or weight loss. The occurrence of exacerbation was then compared to daily mean concentrations of O_3_, SO_2_, NO_2_, PM_10_, and CO obtained from monitoring stations spread across the city. The authors reported a higher relative risk of exacerbation when O_3_ concentration increased by an interquartile range (45.62 μg/m^3^) (Table 1). A significant association was observed for an O_3_ increase 2 days before the onset of exacerbation and a tendency was observed for O_3_ exposure the day prior and the same day as the exacerbation. No statistically significant association was found between other pollutants and the occurrence of exacerbation in patients with CF. Indeed, O_3_ was the only pollutant exceeding national Brazilian standards as well as the WHO air quality guidelines during the study, hence reaching unhealthy levels while other pollutants levels were considered acceptable. However, this study raises several questions as many key elements are not described: the spatial variability of the concentrations of pollutants that allow to differentiate patients; the study of correlations between different pollutants (why are PM excluded from impacting pollutants when they are correlated with NO_2_?); the lack of temporal visibility of pollutant concentrations and the ability to predict the impact on disease on fine time scales; the insufficiently explicit use of clinical data and their very patchy aspect. Also, the physiological importance in defining exacerbation severity differs between criteria (fever vs. >10% decrease of FEV_1_). Overall, a clarification of the positive criteria related to an exacerbation would have provided the necessary information. The discrepancy with Goeminne’s study regarding the impact of PM_10_ and NO_2_ levels may be explained by the methods used for exposure data estimation. Interestingly, levels of PM_10_ and NO_2_ reported by Farhat were higher than Goeminne’s with respective mean concentrations of 42 μg/m^3^ and 103 μg/m^3^ against median concentrations of 24 μg/m^3^ for the two pollutants (Table 1) (Goeminne et al., 2013). These results are consistent with the fact that Farhat’s study was conducted in a highly polluted metropole. However, both studies came to comparable O_3_ concentration. More likely, the absence of association between the increase of PM_10_ and NO_2_ concentration and the occurrence of respiratory exacerbation in the Brazilian study might be explained by the smaller size of the panel but also by the difference in exacerbation definition and inclusion criteria. Goeminne’s definition is restrictive to severe exacerbations because a lot of exacerbations are treated with only oral and/or aerosolized antibiotics. Also, patients who had no exacerbation or no registered use of antibiotic were excluded. Exacerbation definition is less stringent in Farhat’s study which covers a larger panel of CF patients, including patients who did not undertake antibiotic therapy. This may have accounted for a possible not significant impact of other pollutants in Farhat’s study and could suggest that NO_2_ and PM_10_ mainly target airways and favor infection while O_3_ would have a wider impact on other organ function. Finally, patients’ status at the time of the study could also explain the difference observed. Indeed, in Farhat’s study, 70% of CF patients did not present a significant degree of bronchial obstruction and their median Shwachman-Kulczycki score (assessment of CF disease severity based on general activity, physical examination, nutrition and radiological findings) was 75 corresponding to a “Good” clinical condition, while no details on disease severity was provided in Goeminne’s study. Overall, both studies bring additional elements to consider acute exposure to air pollution as negatively impacting the course of CF disease.

## Effects of Air Pollution Exposure in Experimental Models of Cystic Fibrosis

Overall, epidemiological studies on CF patients have highlighted an increased risk of exacerbations, primary infections and reduced lung function associated with air pollution exposure. If these studies identify a negative impact of global air pollution on the course of CF, they however leave remaining questions about how these effects originate. The following chapter is dedicated to present experimental studies designed to answer these questions.

### *In Vivo* Studies

As central contributors to CF airway pathophysiology, the expression of CFTR and airway secretions have been investigated in response to air pollution. A markedly reduced immunofluorescence staining of CFTR was observed in tracheal respiratory epithelium of rats exposed to O_3_ (Qu et al., 2009). A decrease of about 25% in mRNA and protein expression of CFTR and aquaporins 1 (AQP1) and 5 (AQP5) was observed in normal BALB/c mice exposed to diesel particulate matter (Shi et al., 2019). The underlying mechanisms of action of such reduced CFTR expression have not been studied *in vivo*, nor the effect of diesel particulate matter on CFTR channel function. Importantly, the reduced expression of CFTR and aquaporins could result in a decreased cell permeability to water and ions and thus ASL secretion, two key features of CF. Shi et al. (2019) also described an increased Muc5AC mucin expression in BALF in the same mice. Interestingly, although not in the context of CF, O_3_, SO_2_, and particles were found to induce mucous cell metaplasia (Harkema et al., 1999; Sueyoshi et al., 2004), increase mucin expression (Jany and Basbaum, 1991; Cho et al., 1999; Huang et al., 2017) and impair mucociliary clearance and ciliary beating frequency (Pedersen, 1990; Saldiva et al., 1992; Bayram et al., 1998) in rat airway epithelium and human airway epithelial cells. These events would be interesting to consider since they are altered in CF but not yet studied in relation to air pollution exposure in CF models. Unfortunately, no dose-response relationship could be established, as only one dose for O_3_ and diesel particulate matter was used in these animal studies.

*In vivo* studies investigating the effects of air pollution on CF models remain sparse (Table 2), compared to those performed in other models of bronchial disease, such as asthma or COPD. Carbon nanoparticles (CNP), particularly abundant in urban air pollution, has been instilled in Cftr^tm1HGU^ and WT mice by intratracheal route to evaluate their lung inflammatory response (Geiser et al., 2014). The Cftr^tm1HGU^ model is produced by a targeted insertion mutagenesis in exon 10 of the *Cftr* gene (Dorin et al., 1992). It presents a markedly reduced but residual CFTR expression (about 10% of normal mRNA levels) resulting in a mild disease phenotype (Dorin et al., 1994). Of note, this mild airway phenotype is also influenced by genetic background and species differences as compared to humans. CNP instillation provoked, in WT and mutated mice, an acute and transient inflammatory response dominated by neutrophils. This response was maximal the day following exposure. Privileged interaction of NP with airway epithelial cells in mutants due to a reduced clearance of such NP could explain the induction of this inflammatory response. Indeed, Geiser et al. (2014) reported an alteration of titanium dioxide manufactured nanoparticles (TiO_2_NP) cellular uptake in Cftr^tm1HGU^ mice. In WT mice, the cellular uptake of TiO_2_NP is entirely achieved by macrophages while in Cftr^tm1HGu^ mice, 26% of TiO_2_NP are internalized by alveolar epithelial cells—the remaining 74% NP are internalized by macrophages. This suggests a reduced macrophagic clearance of NP in this CF model. These results corroborate those obtained by the same group on inhaled gold NP uptake and cellular localization in transgenic *Scnn1b* mice: compared to WT mice, *Scnn1b* transgenic mice showed a reduced macrophage uptake of gold NP that were mainly localized at the epithelium surface (Geiser et al., 2013). This is of particular interest because this mouse model—characterized by an airway-targeted overexpression of the ENaC *β* subunit—constitutes presently the most complete murine model reproducing human CF pulmonary phenotype (Mall et al., 2004; Zhou et al., 2011). Finally, a reduced airway macrophage phagocytosis of carbonaceous PM has also been described in CF children (Liu et al., 2020), adding to the notion of an overall altered clearance of particulate material in CF disease.

An important feature of CF airway pathogenesis is the excessive and inefficient inflammatory process that could be explained by an altered resolution of inflammation (Roesch et al., 2018; Briottet et al., 2020). The finding that resolution of inflammation differs between Cftr^tm1Hgu^ and WT mice is consistent with this aspect of the clinical description of the CF airway disease. Indeed, at the third day post-instillation, neutrophil count in bronchoalveolar lavages fluid (BALF) is decreased by 70% in WT mice while only by 25% in Cftr^tm1HGU^ mice. BALF pro-inflammatory cytokines levels were also higher in Cftr^tm1HGU^ mice compared to WT for most cytokines tested. As a striking example, G-CSF concentration was 13 times higher in *Cftr* mutant mice, consistent with the slower decline of BALF neutrophils and suggesting a delayed or impaired resolution of inflammation in *Cftr*-deficient mice. The specialized pro-resolving mediators (SPM) which are bioactive lipids triggering the inflammation resolution phase, are thus of particular interest in this context. Interestingly, the levels of 15-hydroxyecosatetraenoic (15-HETE), a metabolite of the lipoxygenase pathway showing anti-inflammatory properties, was significantly lower in *Cftr* mutant mice exposed to CNP (Karp et al., 2004; Ringholz et al., 2014), which is in line with the compromised resolution of CNP-induced airway inflammation in *Cftr* mutants (Geiser et al., 2014). Furthermore, the SPM lipoxin A_4_ (LXA4) was found to be decreased in C57Bl/6 mice in response to cigarette and wood smoke (Awji et al., 2015). SPM also have a therapeutic potential in reversing or preventing pollution-induced inflammation (Kilburg-Basnyat et al., 2018; Lu et al., 2018; Thatcher et al., 2019). Finally, in the airway of CF patients, the biosynthesis of LXA4 and Resolvin D1 (RvD1) SPM are reduced (Karp et al., 2004; Ringholz et al., 2014; Eickmeier et al., 2017; Briottet et al., 2020; Isopi et al., 2020) and related to the sustained levels of pro-inflammatory cytokines and bacterial infection, the altered airway surface hydration and pulmonary function (Verrière et al., 2012; Eickmeier et al., 2017; Codagnone et al., 2018; Ringholz et al., 2018; Isopi et al., 2020). Taken together, these data highlight a possible particular pulmonary susceptibility of CF patients exposed to particulate air pollution; under these conditions, insufficient inflammation resolution could favor the onset of a chronic inflammatory condition and accelerated progression of the CF airway disease.

### *In Vitro* Studies

In non-CF HBE cells, it has been shown that O_3_ decreased CFTR mRNA, protein expression and activity (Qu et al., 2009). This modulation of CFTR expression is dependent of the JAK2/STAT1 pathway which is itself activated by reactive oxygen species (ROS) (Qu et al., 2009). Indeed, ROS inhibitors prevented O_3_-induced CFTR expression decrease by reducing STAT1 phosphorylation. As supporting evidence, a direct decrease of CFTR expression and activity due to oxidative stress has been previously described (Bebok et al., 2002; Cantin et al., 2006a). CFTR internalization or retrograde trafficking to the endoplasmic reticulum as induced by cigarette smoke exposure (Clunes et al., 2012; Marklew et al., 2019) could also be considered to explain the reduced CFTR expression and activity induced by O_3_. In Calu-3 cells, diesel particulate matter (DPM), which contains oxidants, dose-dependently reduced CFTR expression as well as its binding to the plasma membrane (Shi et al., 2019). Although not directly investigated, the authors suggest that previously described mechanisms involving JAK2/STAT1 in reducing CFTR expression could be similarly involved in these effects. Interestingly, the expression of AQP1 and AQP5 expression was also significantly decreased after DPM exposure, while an increased cAMP intracellular content could be detected, potentially promoting CFTR activity. It must be noted that the inhibiting effects of such exposure on CFTR expression and membrane localization were stronger. In another study in Calu-3 cells, CFTR function was found to be reduced by DPM. However, this acquired CFTR dysfunction could be rescued by CFTR potentiator VX-770 (Nguyen et al., 2021). Interestingly, polystyrene NP activated CFTR ion channel in Calu-3 cells (McCarthy et al., 2011). Although not yet studied in CF cells, the modification of CFTR expression after exposure to air pollutants is thus important to consider because the existing low CFTR expression and altered airway fluid secretion in CF patients could be deepen. In class IV–VI CF patients, with residual CFTR function mutations, this could lead to a potential total suppression of CFTR function which would certainly worsen their pathophysiological state.

**FIGURE 2:**
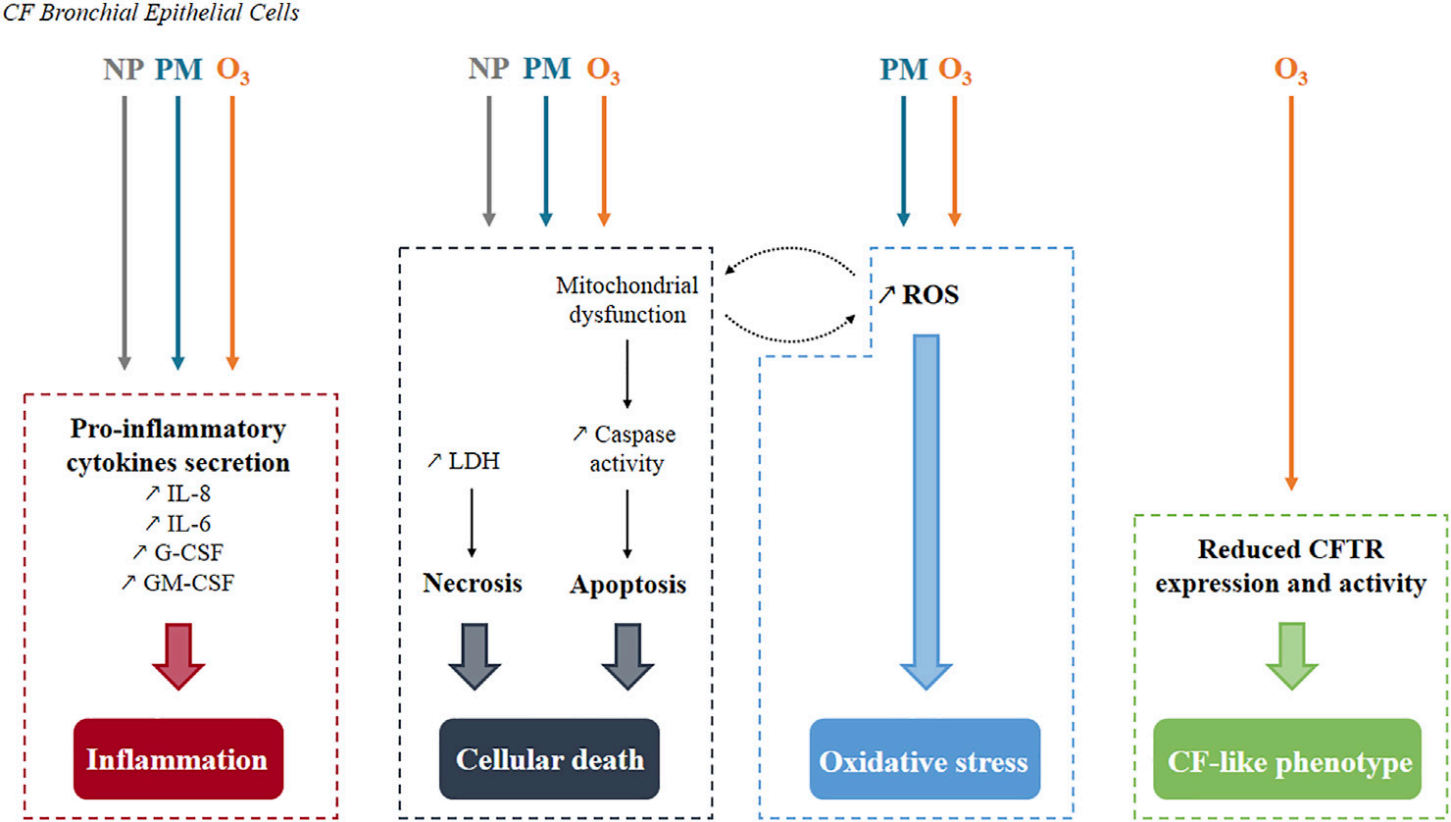
Summary of pollutants’ effects known so far in human CF bronchial epithelial cells. Three common cellular mechanisms have been identified. Inflammation and cell death occur after exposure to either nanoparticles, particulate matter or ozone while oxidative stress is generated only by particulate matter and ozone. Mitochondrial dysfunction and reactive oxygen species production are interconnected as they stimulate each other. Specifically to ozone, a CF-like phenotype is produced with decreased CFTR expression and activity. NP, Nanoparticles; PM, Particulate Matter; O_3_, Ozone; ROS, Reactive Oxygen Species.

Studies published on PM_2.5_/PM_10_, O_3_, and NP exposure effects on CF cells (Table 3) have highlighted three common cellular mechanisms—cell death, oxidative stress, and inflammation (Figure 2). Such effects are not surprising as they are classically described and known to participate in CF pathophysiology (Rosenfeld et al., 2001; Rottner et al., 2007, 2011; Sagel et al., 2012; Voisin et al., 2014; Ziady and Hansen, 2014; Antus, 2016; Roesch et al., 2018) and induced by air pollution exposure (Dagher et al., 2006; Lee et al., 2018; de Oliveira Alves et al., 2020; Gangwar et al., 2020; Li et al., 2020).

**FIGURE 3:**
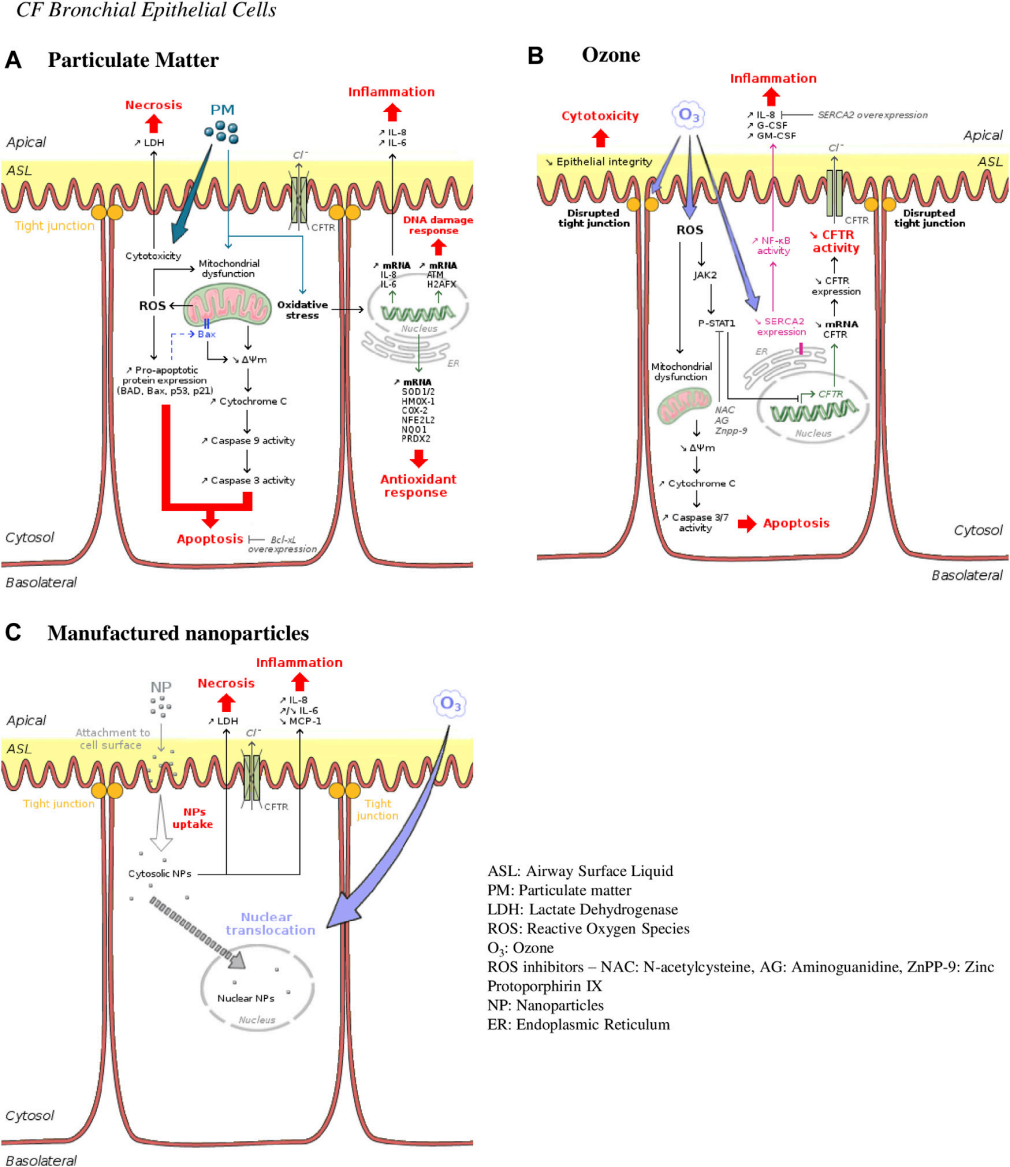
Ambient air pollution mediated effects and mechanisms induced in human CF bronchial epithelial cells. **(A)** Particulate matter effects. Particles are responsible for cellular death occurring either by necrosis or apoptosis. They are also responsible for inflammation, DNA damage and oxidative stress by inducing expression of typical genes involved in such responses—interleukin 8 and 6 (IL-8 and IL-6) for inflammation; histone H2A family member X (H2AFX) for DNA damage repair; superoxide dismutase (SOD1/2), heme oxygenase 1(HMOX-1), nuclear factor erythroid-derived 2-like 2 (NFE2L2) or peroxiredoxin (PRDX2) for antioxidant response. **(B)** Ozone effects. Ozone is directly cytotoxic for bronchial epithelial cells by disrupting tight junctions and epithelial integrity and by causing apoptosis. Promoting the JAK2-STAT1 pathway, ozone leads to pro-inflammatory cytokines secretion through NF-κB activation. CFTR expression is also downregulated leading to CFTR activity decrease and a CF-like phenotype. **(C)** Manufactured nanoparticles effects. Nanoparticles are internalized into bronchial epithelial cells provoking necrosis and inflammation. Co-exposure with ozone causes increase in intranuclear delivery of these nanoparticles.

Cell death occurs either by apoptosis or necrosis or even by both processes in the case of exposure to PM_2.5_ (Kamdar et al., 2008; Leni et al., 2020). Exposure of transformed human CF bronchial epithelial cells (CF HBE cells) or wild-type ones (non-CF HBE cells) to PM_2.5_ (Kamdar et al., 2008) or O_3_ (Ahmad et al., 2011) induced an increase in DNA fragmentation, propidium iodide staining, caspase 3/7 and 9 activity and an increased expression of pro-apoptotic mediators (Figures 2, 3). Of note, this increase was greater in CF HBE cells suggesting that these cells are more sensitive to PM_2.5_ and O_3_ than non-CF ones. While the level of anti-apoptotic proteins remained unchanged with exposure, the overexpression of the anti-apoptotic Bcl-xL contributes to significantly reduce apoptosis in CF HBE cells exposed to PM_2.5_. Mitochondrial dysfunction seems to be at the origin of such apoptosis (Kamdar et al., 2008; Ahmad et al., 2011). Indeed, a decrease of mitochondria membrane potential, of cytochrome C release and pro-apoptotic Bcl-2 associated X (Bax) translocation to mitochondria were reported upon exposure to PM_2.5_ and O_3_. Mitochondria-dependent generation of reactive oxygen species (ROS) was induced by exposure to PM_2.5_ only (Kamdar et al., 2008). Likewise, baseline production of ROS was higher in CF HBE cells revealing increased oxidative stress in CF cells. By promoting further mitochondrial dysfunction, ROS generation maintains oxidative stress, which could be responsible for the higher baseline apoptosis observed in CF cells. Furthermore, as a marker of cytotoxicity, increase lactate dehydrogenase (LDH) release from damaged cells has been linked to necrosis in CF cells exposed to silver (AgNP) and carbon manufactured NP (Jeannet et al., 2016). Also, a dose-dependent increase in LDH release was observed after exposure of normal and CF HBE cells to PM_2.5_ and PM_10_ (Leni et al., 2020), aged gasoline exhaust particles (Künzi et al., 2015) or wood combustion particles (Krapf et al., 2017). A loss of transepithelial resistance was showed in CF cells exposed to O_3_ suggesting the disruption of tight junctions (Ahmad et al., 2011). However, these NP did not interfere with epithelial integrity as intact apical junctional complexes were reported after such exposure (Ahmad et al., 2012; Jeannet et al., 2016). The observed modification of tight junction properties after O_3_ but not NP exposure indicates a more pronounced susceptibility of CF cells to O_3_ than to NP. Yet, CF HBE cells sensitivity to NP could be potentiated in presence of O_3_. Indeed, after cell surface attachment, NP are mainly internalized in cytosol but this localization was partly switched to nucleus when cells were co-exposed to O_3_ thus giving NP a potential to exert further harmful effects (Ahmad et al., 2012).

Oxidative stress response has been shown to be stronger in CF HBE cells primary cultures compared to non-CF HBE cells. Wood combustion particles upregulated the expression of the oxidative stress markers heme oxygenase 1 (HMOX-1) and cyclooxygenase 2 (COX-2) (Krapf et al., 2017). PM_2.5_ and PM_10_ also upregulated genes involved in oxidative stress (superoxide dismutase one and 2—SOD1 and 2), antioxidant defense (HMOX-1, nuclear factor erythroid-derived 2-like 2—NFE2L2), oxidative protection (NADP(H) quinone dehydrogenase—NQO1, peroxiredoxin—PRDX2) as well as genes involved in DNA damage repair (ataxia telangiectasia mutated kinase—ATM, histone H2A family member H2AFX) (Leni et al., 2020). This oxidative/anti-oxidative imbalance could yield to an inflammation cascade. Indeed, IL-8, G-CSF, and GM-CSF levels were found to be markedly increased following O_3_ exposure in CFBE41o- cell line (Ahmad et al., 2011). O_3_-mediated inflammation is dependent on the NF-κB signaling pathway modulated by SERCA2 (Sarcoendoplasmic Reticulum Calcium ATPase). SERCA2 is downregulated by O_3_ and the specific downregulation of SERCA2 activates NF-κB pathway and the subsequent IL-8 release (Ahmad et al., 2011). The O_3_-induced IL-8 secretion was reversed by SERCA2 overexpression. Interestingly, SERCA2 expression is constitutively decreased in CF airway epithelia, which could potentially explain the basal inflammation observed in these cells (Ahmad et al., 2009). Indeed, basal IL-8, G-CSF, and GM-CSF levels were greater in CF airway epithelial cell lines and CF primary cultures than in non-CF cells (Ahmad et al., 2011) as well as IL-6 and MCP-1 levels which are, in CF cells, constitutively largely exceeding those of non-CF cells (Künzi et al., 2015; Jeannet et al., 2016). Such higher pro-inflammatory cytokines levels could also participate in turn to ROS generation. As regard to cytokine secretion, the effects of particle exposure are more controversial. Indeed, in both non-CF and CF HBE cells IL-8 levels increased after exposure to PM (Leni et al., 2020), AgNP and CNP (Jeannet et al., 2016) while polystyrene NP did not induce modification of IL-8 release (Ahmad et al., 2012). IL-6 levels either increased in response to PM, AgNP, and CNP in normal and CF HBE cells but decreased after exposure to aged gasoline particles in CF HBE cells. Upon exposure to PM and NP, MCP-1 levels increased in normal cells while they decreased in CF HBE cells (Künzi et al., 2015; Jeannet et al., 2016; Leni et al., 2020). Wood combustion particles did not significantly alter any cytokine release in any of the cell model considered (Krapf et al., 2017).

## Knowledge Gaps and Future Directions

Unfortunately, there is no epidemiological data on CF prior to the industrial revolution that could bring to light the influence of air pollution composition on CFTR or on the CF airway disease. Indeed, anthropological studies suggests that CFTR mutation arose in the early Bronze Age and that the mutations spread from west to southeast Europe during ancient migrations *via* healthy carriers (CF is an autosomal recessive disease—people with CF inherit one mutated copy of the *CFTR* gene from each parent, referred to as a carrier). However, the first description of the CF disease dates only back to the 1930s (Andersen, 1938). In the 1950s, the median life expectancy for patients with CF was a few months, not allowing any conclusion to be drawn regarding the impact of air pollution. Finally, the causative CFTR gene was only discovered 30 years ago (Riordan et al., 1989). There are geographic differences in life expectancy of CF patients which could be related to different air pollution, but these discrepancies are mostly related to the status of developing country or not, where the weight of accessibility to treatments is bigger than that of air pollution. Substantial increased number of adults with CF in developed countries has resulted from delivery of care in well-organized, multidisciplinary CF centers and the use of effective drugs to treat infection and improve mucociliary clearance (Castellani et al., 2009; McCormick et al., 2010). In contrast, in countries with less well-resourced health-care systems, median life expectancy can be in the second decade of life because of the lack of access to treatment.

Epidemiological studies provide information on the global effect of air pollution on the course of CF disease. However, individual exposure is difficult to follow and only few pollutants are currently explored. Exposure estimation is tricky. Except one study which used spatial interpolation model (Goeminne et al., 2013), all the others estimated individual exposure on average measures obtained by the closest oriented-population monitoring air stations rather than direct monitoring at the patient’s residence. As a consequence, possible exposure misclassification could occur. Monitoring stations are mainly found in urban areas. How accurately assess exposure in rural areas? In addition, only few pollutants are monitored, but are they representative of the atmospheric mixture? Actual individual exposure also depends on factors like time spent outdoors, distance from pollution sources and weather patterns. For example, meteorological conditions are of primary importance because of their pivotal link to both CF and pollution: they have effects on CF clinical condition—as shown for temperature (Collaco et al., 2011) and season (Psoter et al., 2013) on *P.aeruginosa* lung infection—and they also influence on pollution chemical composition and evolution. Therefore, the inclusion of meteorological variables should be considered. A correct exposure estimation is also in relation with an accurate determination of possible confounders. In most epidemiological studies reviewed here, adjustment was made on socio-economic status. People of low socio-economic status tend to live in areas with higher ambient air pollution, have lower income and thus limited access to health care which could explain poorer health outcomes. Adjustment on socio-economic status may have reduced residual confounding due to these variables. However, these studies reveal the lack of inclusion of other confounding exposures such as tobacco smoke. Of note, cigarette smoking exposure has been associated with worse clinical prognosis in CF—decreased growth and weight (Rubin, 1990; Kovesi et al., 1993; Kopp et al., 2015), smaller lung function (Kovesi et al., 1993; Smyth et al., 1994; Collaco et al., 2008), increased colonization by MRSA (Kopp et al., 2015), longer periods of intravenous antibiotic treatment (Gilljam et al., 1990), airway surface dehydration (Clunes et al., 2012) and CFTR dysfunction (Cantin et al., 2006b; Raju et al., 2013; Marklew et al., 2019; Rasmussen et al., 2020). In a very recent study, tobacco smoke has also been shown to limit the therapeutic benefit of tezacaftor/ivacaftor therapy in pediatric CF patients (Baker et al., 2021). Similar limiting effects of air pollution on such treatments could be considered in CF patients. On the opposite, the effects of cigarette smoke exposure were reversed with the use of the CFTR potentiator ivacaftor (VX-770) in non-CF HBE cells (Sloane et al., 2012; Raju et al., 2017). One could thus also hypothesize the potential prevention of air pollution effects by CFTR modulators as shown with DPM-induced CFTR dysfunction rescue by VX-770 (Nguyen et al., 2021). Tobacco smoke and air pollution co-exposure must be considered in the context of worsening exacerbations and lung function. The important point here is that air pollution is part of a wider concept called exposome. Exposome corresponds to the sum of all environmental exposures an organism experiences throughout his/her life, from conception to death (Wild, 2012). This concept emphasizes that it is impossible to know to what extent air pollution exposure only influences CF phenotype as one individual is never exposed to air pollution alone. Environmental exposures more likely interact with each other and have cumulative effects. That is why, to be as realistic and accurate as possible, air pollution should be considered as part of a complete and complex exposome to reduce potential residual confounding. In addition to the complexity of this external exposome—all the external exposures—comes the complexity of an internal exposome—all the biological responses to exposures—that takes its origin, in the context of CF, in the great diversity of CF mutations and their respective consequences on the pleiotropic functions of CFTR itself. Keeping in mind this notion of exposome is of the utmost importance in developing new experimental concepts and lessen the misinterpretation of data. This is true for epidemiological studies as well as for experimental ones. Indeed, studying environmental exposures on animals maintained in strictly controlled conditions—i.e., same exposome—can only provide approximative indications on the potential biological responses in human populations who are genetically heterogenous, live in different environments, have different occupations, etc. and thus have complex and divergent internal and external exposomes (Bolon and Haschek, 2020).

Experimental studies use animal and cellular models. CF animal models are numerous and different based on the nature of the mutation in the *Cftr* locus (Wilke et al., 2011). In mice, most of the knock-out models present a severe mortality due to intestinal obstruction which limits the possibility to study the disease at adult age. F508del mutation models have a good survival until maturity and have the advantage to reproduce the most common mutation found in patients. However, they mainly reproduce the intestinal aspect of the disease with no basal specific lung phenotype. On the opposite, the *Scnn1b* transgenic model presents a CF-like lung disease but has no intestinal defect. In addition, this model is not mutated in the *Cftr* gene thus diverting the origin of CF disease and limiting other possible effects resulting from *Cftr* mutations. Apart from mice, other CF animal models developing a “spontaneaous” lung disease have emerged such as pigs, ferrets or rats (Semaniakou et al., 2019). As for CF animal models, there is a wide range of existing CF epithelial cell models (Castellani et al., 2018). The airway epithelium is characterized by a wide diversity of cell types: basal cells, ciliated cells, goblet cells, club cells, ionocytes and more (Montoro et al., 2018). These different cell types could have different susceptibility to air pollution. In the specific context of CF, ionocytes—cells with a high level of CFTR expression (Plasschaert et al., 2018; Scudieri et al., 2020)—could be particularly interesting to examine. Yet, this cellular complexity of the respiratory epithelium is not always considered. Indeed, transformed or immortalized cell lines constitute a simplistic single barely differentiated cell type model. On the other hand, fully differentiated primary HBE cells cultured at air-liquid interface, despite subjected to donors’ variability, more closely mimic the *in vivo* lung environment and therefore relevant exposure conditions. These differences in model construction could explain why responses might be very different from one study to another.

Beside the limits of the different animal and cellular models of CF exposed above, another major limitation of the experimental studies reviewed here resides in pollutants consideration. Several challenges remain to be tackled. As mentioned earlier, air pollution consists of a complex reactive mixture evolving in space and time. Yet only few pollutants—used as indicators and representing a tiny part of the atmospheric mixture (O_3_, NO_2,_ SO_2_, PM_2.5_, and PM_10_)—are currently monitored. As an example, ultrafine particles (PM_0.1_) are not measured directly nor subjected to guideline values while causing deleterious health effects (MacNee and Donaldson, 2003; Schraufnagel, 2020). Moreover, many toxicological studies have been based on the study of individual pollutants. As a primary approach, studying individual pollutants is interesting for mechanistic insight and to give a first glimpse of their respective toxicity. However, it is limited when numerous pollutants interact together. One isolated pollutant will not necessarily have the same effects alone or in combination with others. The relevance of existing experimental studies can therefore be largely challenged based on the absence of consideration of such synergism and reactivity between pollutants making it thus difficult to extrapolate results to “real life.” In this context of considering pollutants’ dynamic, few studies exist on pulmonary effects of secondary or aged aerosols (Delfino et al., 2010; Gaschen et al., 2010; Rager et al., 2011; Diaz et al., 2012; Künzi et al., 2013; Künzi et al., 2015; Krapf et al., 2017). Ahmad et al. are to our knowledge the only ones to have combined NP and O_3_ exposition, a first step toward consideration of multi-pollutant interactions (Ahmad et al., 2012). A disruptive approach consists to reproduce multiphasic chemical processes in the laboratory—i.e., continuously produce environments representative of urban atmospheres—in order to study the impact of air pollution in particular in the multi-pollutant synergy and multiphasic dimensions (Coll et al., 2018). For example, the authors have developed a platform, named PolluRisk, which allows to reproduce for days the atmospheric phases (gases and aerosols) similar to those of cities like Paris or Beijing for example. It is resulting from the coupling of an atmospheric simulation chamber with exposure devices able to host preclinical models. The experimental protocol consists in the continuous injection of primary pollutants (NOx, organic compounds mix simulating anthropogenic emissions, SO_2_, soot, inorganic salts and potentially mineral dust particles if needed) at low concentrations (ppb levels in air) in the chamber. Due to the residence time in the atmospheric simulation chamber—around 4 h to represent air masses at a regional scale—the mixture is exposed to an artificial solar irradiation, allowing secondary pollutants such as O_3_, NO_2_, formaldehyde, and peroxyacetyl nitrate (PANs) as well as complex polyfunctional organics including Secondary Organic Aerosol (SOA) to be formed. This way, they can reach their chemical steady state. Exposure devices are then “fed” with a constant flow of such a mixture. A challenge of such chambers is to reach realistic pollutants doses, a key point as *in vivo* and *in vitro* studies usually expose animal models and cells to non-realistic elevated pollutants concentrations. For example, in the studies reviewed here (Tables 2, 3), an O_3_ exposure from 100 ppb to 1.5 ppm corresponds to 195–3 000 μg/m^3^, levels far from the WHO guidelines values—100 μg/m^3^ 8-h daily maximum and 60 μg/m^3^ 8-h mean (World Health Organization, 2021)—and not reached in real life. For particulate exposure, doses are usually more realistic but mostly match a high-exposure scenario.

## Conclusion

A complex relationship exists between gene and environment into determining human disease phenotypes. If CF is definitely caused by a genetic defect, environmental aggressions most probably participate to the progression of CF airway disease. Epidemiological studies provide some data for a possible contribution of air pollution to CF airway severity and morbidity. In addition and although scarce, the existing experimental literature supports this possible contribution of air pollution to CF airway disease and gives an insight for possible mechanisms involved. However, it also underlies limitations, both conceptual—missing the complexity of exposome considerations—and experimental—linked to the use/evaluation of one pollutant at a time. For sure these questions need to be addressed in future research.
